# Endoplasmic reticulum homeostasis in plant–pathogen interactions: new scenarios for an old story

**DOI:** 10.1093/jxb/erae404

**Published:** 2024-09-21

**Authors:** Yuhan Liu, Shiping Tian, Tong Chen

**Affiliations:** State Key Laboratory of Plant Diversity and Specialty Crops, Institute of Botany, Chinese Academy of Sciences, Beijing 100093, China; China National Botanical Garden, Beijing 100093, China; University of the Chinese Academy of Sciences, Beijing 100049, China; State Key Laboratory of Plant Diversity and Specialty Crops, Institute of Botany, Chinese Academy of Sciences, Beijing 100093, China; China National Botanical Garden, Beijing 100093, China; University of the Chinese Academy of Sciences, Beijing 100049, China; State Key Laboratory of Plant Diversity and Specialty Crops, Institute of Botany, Chinese Academy of Sciences, Beijing 100093, China; China National Botanical Garden, Beijing 100093, China; Bielefeld University, Germany

**Keywords:** Cell death, endoplasmic reticulum, ER-phagy, pathogen, plant, unfolded protein response

## Abstract

The endoplasmic reticulum (ER) is a specialized organelle that connects almost all subcellular structures from the plasma membrane to the nucleus. The ER is involved in secretory protein synthesis, folding, and processing. Evidence has emerged that the ER is at the frontier of the battle between plant hosts and pathogens. Its structural and functional homeostasis is crucial for the survival of plant cells. Pathogens secrete effectors to take over normal functions of the ER, while host plants fight back to activate ER stress responses. Exciting advances have been made in studies on host plant–pathogen dynamics during the past decades, namely some new players involved have been recently resolved from both pathogens and hosts. In this review, we summarize advances in identifying structural characteristics of the key pathways and effectors targeting the ER. Newly identified ER-phagy receptors and components downstream of inositol-requiring 1 (IRE1) will be described. Future studies will be envisaged to further our understanding of the missing parts in this dynamic frontier.

## Teaser

This review summarizes the latest advances in the control of endoplasmic reticulum homeostasis during pathogen infection, and proposes some directions to be addressed in the future.

## Introduction

The endoplasmic reticulum (ER) serves as a highly dynamic organelle that is crucial for cell vitality and communications with the surrounding environment (Wang *et al.*, 2017; Stefano and Brandizzi, 2018). It is not merely the place where lipids and proteins are synthesized, but also the site of protein glycosylation and the gateway into the secretory pathway for nascent proteins (Taiz and Zeiger, 2002). Therefore, it is an organelle essential for eukaryotic cells to endure biotic and abiotic stress. When cells are subjected to persistent stress conditions, unfolded or misfolded proteins accumulate within the ER, culminating in so-called ER stress, while cells can mitigate this ER stress and maintain cellular homeostasis by eliminating unfolded or misfolded proteins via the ER quality control (ERQC) machinery (Pastor-Cantizano *et al.*, 2020; Brandizzi, 2021; Ko and Brandizzi, 2024).

**Fig. 1.**
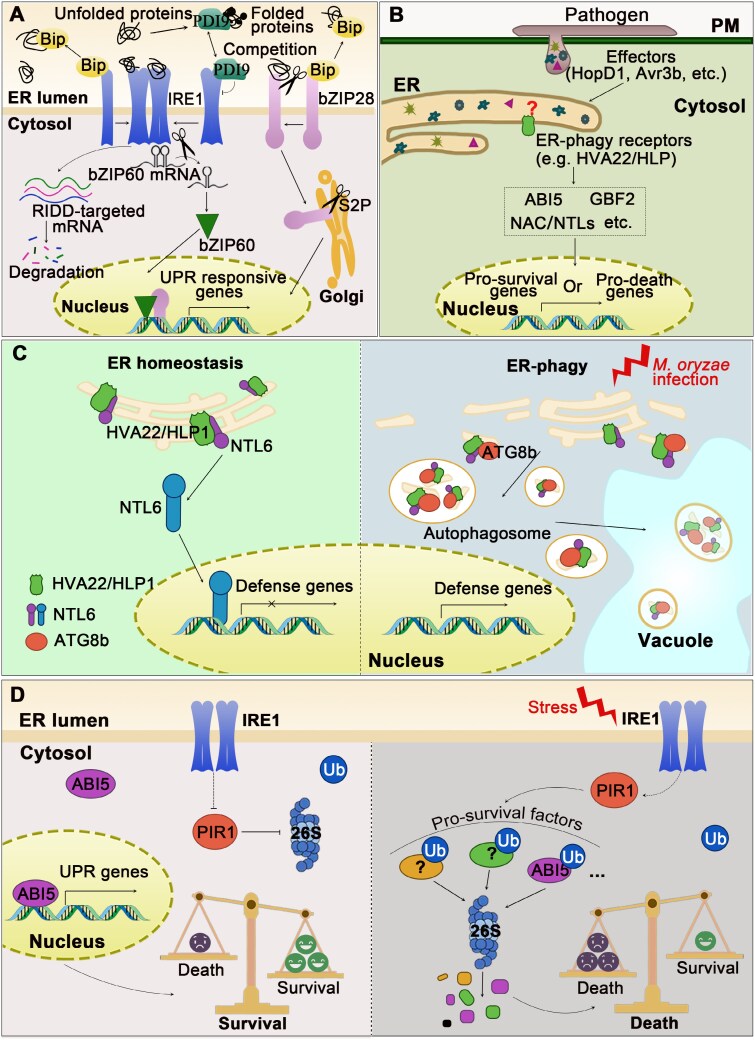
Mechanisms underlying the maintenance of structural and functional homeostasis of the endoplasmic reticulum (ER) in plants. (A) IRE1, bZIP60, and bZIP28 make up the core components. In plants, inositol-requiring enzyme (IRE)1A and IRE1B are maintained in an inactive state as they bind to binding protein (BiP), while excessive misfolded or unfolded proteins trigger the dissociation of BiP from IRE1, thereby activating the RNase domain of IRE1. Subsequently, IRE1 catalyzes the splicing of bZIP60 mRNA and produces bZIP60, and the selective degradation of mRNAs [regulated IRE1-dependent decay (RIDD)]. Recently, Carrillo *et al.* (2024) reported that protein disulfide isomerase 9 (PDI9) could modulate the UPR through two competing activities: secretory protein folding and interaction with IRE1 to maintain proteostasis in plants. Alternatively, excessive misfolded or unfolded proteins also cause the dissociation of BiP from bZIP28, thus triggering its translocation to the Golgi apparatus. bZIP28 is further cleaved first by S1P/S2P, although the machinery is still under debate. The resulting active transcription factor (TF) is translocated to the nucleus. (B) Pathogens secrete effectors that interfere with normal functions of the ER via various targets. It has been known that ER-phagy receptors (such as HVA22 family members) modulate downstream signaling by activating cleavage of membrane-tethering NACs/NTLs, which ultimately migrate into the nucleus to trigger pro-survival or pro-death transcriptional responses. (C) OsHLP1, a member of the HVA22 family, functions as an ER-phagy receptor in rice (*Oryza sativa* L.) by interacting with OsATG8b and recruiting ER subdomains and the cargo protein OsNTL6 to autophagosomes upon infection, eventually activating the resistance of rice to *Magnaporthe oryzae* (Meng *et al.*, 2022; Liang *et al*., 2023). (D) Ko and Brandizzi (2022) proposed that the IRE1–proteasome system signaling cohort controls cell fate determination under ER stress. Under non-ER stress conditions (left panel), ABI5 functions as a pro-survival activator of the UPR by directly inducing the expression of UPR-related genes, such as *bZIP60* and *BiP3*. Upon ER stress (right panel), PIR1, as a pro-death E3 ligase, represses the stability of pro-survival factors (including ABI5 and possibly other TFs) in the UPR, playing a critical role in IRE1-dependent cell fate determination. Arrows represent positive regulation; T-bars represent negative regulation; a question mark indicates an unknown target; and the dashed lines represent indirect or unknown mechanisms.

It has been revealed that pathogen invasion and colonization may lead to a rapid transcriptional reprogramming of many genes in host plants, encompassing increased biosynthesis of pathogenesis-related (PR) proteins or pattern recognition receptors (PRRs). This may result in substantial accumulation of unfolded or misfolded proteins (Shan *et al.*, 2008; Nekrasov *et al.*, 2009). When the quantity of unfolded proteins surpasses the capacity of the folding machinery, the ER may reduce *de novo* protein synthesis, up-regulate the expression of chaperone proteins, and eliminate misfolded proteins, which are characteristic of the activation of the unfolded protein response (UPR) (Bao and Howell, 2017; Carrillo *et al.*, 2024) (Fig. 1A). In addition to ER-associated protein degradation (ERAD) that degrades misfolded proteins via the ubiquitin–proteasome system (UPS), ER-phagy establishes an alternative route. However, prolonged or severe ER stress may have proteotoxic consequences and result in programmed cell death (PCD). All these intricate mechanisms contribute significantly to the final outcome of host–pathogen interaction (Deng *et al.*, 2011; Houck *et al.*, 2014; Verchot and Pajerowska-Mukhtar, 2021; Ko and Brandizzi, 2024). This review will encapsulate some recent findings reported for both sides of this arm race to determine cell fate (briefly summarized in Box 1), and envisage directions to be further addressed in the future.

## Pathogens secrete effector proteins targeting the ER to manipulate normal ER functions

### Effectors targeting the ER carry a conserved C-terminal tail-anchor domain

Filamentous fungi secrete apoplastic and/or cytoplasmic effectors, thereby infecting an extensive array of economically important crops (Jing and Wang, 2020). In the effector repertoires of *Pythium* and *Phytophthora* species, Arg–X–Leu–Arg (RXLR) effectors have been comprehensively recognized, comprising an N-terminal signal peptide responsible for protein secretion and RXLR/EER (Asp–Glu–Glu–Arg) motifs essential for subsequent internalization by endocytosis (Dou *et al.*, 2008; Wang *et al.*, 2023). Some proteins that contain the C-terminal Lys–Asp–Glu–Leu (KDEL) sequence can be recognized by the KDEL receptor, and be retrieved from post-ER compartments and returned to the ER through a retrograde translocation in COPI-coated vesicles (Wu *et al.*, 2020; Praznik *et al.*, 2022). Recently, it was hypothesized, and subsequently substantiated by experiments, that a conserved C-terminal transmembrane domain (TMD) or tail-anchor domain may govern the targeting of effector proteins from downy mildew to host ER (Breeze *et al.*, 2023). Based on the protein topology characteristics, the authors devised a bioinformatic pipeline to predict ER-localized effectors in the effectorome of *Phytophthora infestans*, which may be instrumental in identifying effectors targeting the ER in other pathogens. Furthermore, Wang *et al.* (2021) identified a variable domain I translocation signal (VDIT) within the variable domain of nematode CLAVATA3/EMBRYO SURROUNDING REGION (CLE)-like effector proteins, which usurps the ER secretory pathway, which was conserved among three phytonematode genera in Arabidopsis and tobacco. However, the mechanism underpinning the post-translational trafficking from cytoplasm to apoplast necessitates further elucidation (Wang *et al.*, 2021). Although the results were relevant, it should be noted that the findings were reported in nematodes rather than pathogens.

### Emerging effectors targeting proteins of the ERQC mechanism

The identification of effectors targeting the ER that specifically manipulate the ER has indicated their diverse modes of action in subduing host immunity (Jing and Wang, 2020) (Fig. 1B). Notable among them is Pi03192, which impedes the nuclear translocation of NTP1 and NTP2 to activate plant immunity (McLellan *et al.*, 2013), PsAvh262 stabilizes BiPs from degradation by the 26S proteasome, thus facilitating the colonization of *Phytophthora sojae* (Jing *et al.*, 2016). Furthermore, PcAvr3a12 suppresses the activity of FKBP15-2, inhibiting the immunity probably provided by increased protein secretion capacity (Fan *et al.*, 2018). It was recently disclosed that AVR_A1_ and BEC1016, two powdery mildew effectors, specifically targeted the ER J-domain protein *Hv*ERdj3B in barley (Li *et al.*, 2024). The J-domain proteins are related to the bacterial DnaJ chaperones, also known as heat shock proteins 40 (HSP40). They interact with HSP70 proteins, such as BiPs in the ER, to which they deliver misfolded client proteins and stimulate the HSP70 ATPase activity (Fatima *et al.*, 2021). The binding of these effectors to ERdj3B could decrease the protein folding capacity of the ER, further suppressing the plant immune response. AVR_A1_ has been previously demonstrated to be directly recognized in the cytosol by Mildew locus A 1 (MLA1), the nucleotide-binding leucine-rich-repeat (NLR) receptor (Lu *et al.*, 2016). These two effectors could both target *Hv*ERdj3B; however, it is noteworthy that the post-translational uptake of both effectors may rely on the Sec61 translocon complex, but be independent of the signal peptides of both effector proteins, suggesting that *Blumeria hordei* may hijack Sec61 via an as yet unidentified mechanism to facilitate infection (Li *et al.*, 2024).

### ER stress in pathogens

This ER stress arises when pathogens deliver effector proteins or secondary metabolites (such as mycotoxins) via the ER secretory pathway. Hence, they potentially encouter ER stress imposed by host plants, affecting penetration. BcSfb3, a Sec24p homolog, is implicated in ER to Golgi transport of proteins in *Botrytis cinerea*. Recently, it was found that this protein orchestrates ER-phagy independent of its role in secretion. The Δ*BcSfb3* mutant showed reduced pathogenicity in host plants and higher sensitivity to ER stress, whilst *BcSfb3* deletion caused UPR and ER-phagy (Wang *et al.*, 2024). Another insightful study also illustrates the crucial roles of ER homeostasis in fungal aggressiveness. Heimel *et al.* (2013) identified the homologs of Hac1 (for Homologous to ATF/CREB1) and IRE1 (inositol-requiring 1) in *Ustilago maydis*, further demonstrating that the UPR was intricately linked to the b mating type-dependent signaling. The C-terminus of the Hac1 homolog, Cib1 (for Clp1 interacting bZIP1), was responsible for the interaction with the b target Clampless1 (Clp1). This molecular interplay further stabilized Clp1 and enhanced the ER stress tolerance of *U. maydis*. Moreover, the UPR facilitated the proliferation of *U. maydis in planta* by dephosphorylating the pheromone-responsive mitogen-activated protein kinase (MAPK) Kpp2, ultimately suppressing mating and formation of infectious filaments (Schmitz *et al.*, 2019). These findings imply that the maintenance of ER homeostasis of pathogens is critical for their successful colonization. Conversely, plants might exploit this mechanism to fend off pathogens.

## Host plants employ ER stress sensors and ER-phagy to alleviate unfolded protein responses

### ER-phagy receptors

Autophagy constitutes an evolutionarily conserved pathway that sequesters cytoplasmic substances or damaged organelles into autophagosomes, which subsequently fuse to lysosomes or vacuoles for degradation (Houck *et al.*, 2014; Bao *et al.*, 2018). ER-phagy refers to the autophagy-driven elimination of the ER, thereby protecting cells from damage caused by excessive ER stress (Liang *et al.*, 2023).

The majority of the reported ER-phagy receptors in plants are integral ER membrane proteins, such as ATG8-interacting protein 1 (ATI1), reticulon 1 (RTN1), and ROOT HAIR DEFECTIVE3 (RHD3) (Zhang *et al.*, 2020; Wu *et al.*, 2021; Sun *et al.*, 2022). Nevertheless, some receptors may have an alternative cellular localization. As an ER-phagy receptor conserved in both plants and mammals, C53 is a cytosolic protein interacting with ATG8, but this interaction depends on the non-canonical ATG8-interacting motif (sAIM), rather than the canonical AIM (Stephani *et al.*, 2020). It was recently reported that OsHLP1, a member of the HVA22 family, maintained ER homeostasis by ER-phagy in rice (*Oryza sativa*) plants (Fig. 1C). OsHLP1 overexpression disrupted the normal ER network in rice cells (Meng *et al.*, 2022). Similarly, this protein interacted with OsATG8b via its LIR motifs and IDR domain to activate ER-phagy, thereby targeting OsNTL6 for autophagic degradation upon *Magnaporthe oryzae* infection. As OsNTL6 functioned as a negative regulator of resistance, this ER-phagy activity augmented the resistance of rice seedlings against *M. oryzae*. Notably, AtHVA22J, an OsHLP1 ortholog in *Arabidopsis thaliana*, also exhibited analogous function by stimulating ER-phagy, suggesting a substantially conserved function of HVA22 family proteins across the plant kingdom. These newly identified ER-phagy receptors highlight an essential role for HVA22-family proteins upon ER stress (Liang *et al.*, 2023). The OsHLP1–OsATG8b–OsNTL6 module contributes to immune activation by restoring functional homeostasis of ER via ER-phagy and alleviating the repression on defense genes.

### New UPR regulators functioning via IRE1 signaling

Upon encountering ER stress, plants rapidly trigger the UPR as a protective mechanism to restore ER homeostasis (Pastor-Cantizano *et al.*, 2020). Four pathways are implicated: (i) augmentation of ER chaperone synthesis for protein folding; (ii) up-regulation of lipid synthesis to expand ER capacity; (iii) blockage of global translation to control protein loading into the organelle; and (iv) up-regulation of ERAD genes to mitigate unfolded protein accumulation in the ER lumen (Nawkar *et al.*, 2018; Pastor-Cantizano *et al.*, 2020; Simoni *et al.*, 2022). The UPR is primarily orchestrated by two signaling cascades. In addition to the signaling mediated by bZIP28 upon its translocation from the ER membrane to the nucleus, IRE1, a dual-functioning protein kinase and RNase, is activated upon exposure to ER stress (Li and Howell, 2021). IRE1 cleaves the *bZIP60* transcript, and the product after cleavage retains a nuclear localization signal. The nascent bZIP60 further transcriptionally modulated the genes associated with the ER stress response (Ko and Brandizzi, 2024).

Despite the identification of the functions of IRE1 in the UPR, the mechanism for its involvement in the ultimate survival or death decision remains elusive. Recently, in an endeavor to identify the *ire1a/b* lethality suppressor in Arabidopsis, Ko *et al.* (2023) reported that PHOSPHATASE TYPE 2CA (PP2CA)-INTERACTING RING FINGER PROTEIN 1 (PIR1), a plant-specific E3 ligase, functioned to fine-tune the UPS and UPR equilibrium downstream of IRE1 (Fig. 1D). *PIR1* loss of function stabilized ABI5, which directly activated the expression of *bZIP60*, thereby triggering transcriptional reprogramming and enhancing the pro-survival UPR. Indeed, previous studies have reported some E3 ligases having a role in ERAD, which functioned as a pro-survival UPS to alleviate proteotoxic stress in the ER (Liu *et al.*, 2021). This study further addressed that the UPS was necessary to instigate cell death upon unmitigated ER stress, when PIR1 repressed the stability of pro-survival ABI5 in the UPR as a pro-death UPR effector downstream of IRE1a/b (Ko *et al.*, 2023). This study underscores the crucial role of IRE1 in making the survival or death decision in the case of ER stress. However, the precise mechanism by which IRE1 modulates PIR1 remains unexplored. It would be intriguing to investigate whether this machinery operates in other species and whether PIR1 may have additional substrates for ubiquitination.

It should be noted that efficient transcriptional reprogramming is essential in dealing with ER stress. Therefore, rigorous regulation of the master transcription factors (mTFs), such as bZIP28 and bZIP60, in ER stress is imperative. Ko and Brandizzi (2022) identified the abscisic acid (ABA)-associated regulator G-class bZIP transcription factor 2 (GBF2) as a transcriptional repressor of the UPR. By competing with the mTFs at the G-box motif, GBF2 repressed the expression of UPR genes. Conversely, a *gbf2* null mutation enhanced UPR gene expression and suppressed the lethality of a *bzip28 bzip60* mutant in unmitigated ER stress (Ko and Brandizzi, 2022). Moreover, ABA biosynthesis modules have been identified in the *B. cinerea* wild-type strain TBC-6 from wheat stems and leaves, and later in a series of engineered mutants (Ding *et al.*, 2016). Efforts should be directed towards determining whether *B. cinerea* might exploit ABA biosynthesis and signaling to interfere with the normal operations of the UPR and thereby achieve infection of host plants. Recently, in a study to screen for natural variations in ER stress sensitivity among 350 *A. thaliana* accessions, Pastor-Cantizano *et al.* (2024) identified BON-ASSOCIATED PROTEIN2 (BAP2), a regulator of PCD, as a negative regulator under UPR sufficiency, while BAP1 and BON1 were not involved. Intriguingly, BAP2 induced the expression of NAC089 as a pro-death regulator under UPR insufficiency. These findings provide empirical evidence for the underlying mechanisms by which the UPR triggers PCD when ER stress is unresolved. The authors reported an unexplored function of BAP2 as a rheostat for pro-survival and pro-death signaling alongside IRE1, while further endeavors are still needed to dissect the regulatory mechanism of BAP2 (Pastor-Cantizano *et al.*, 2024).

### Reactive oxygen species, a traditional yet intricate player in ER stress

As an oxidized milieu relative to other cellular compartments, the ER is also a post-translational modification site for oxidative folding linked to disulfide bond formation (Eletto *et al.*, 2014). Recent studies have revealed the roles of the redox status in ER stress resilience and UPR induction. Transgenic tomato plants overexpressing protein disulfide isomerase (PDI) exhibited enhanced antioxidant capacity as compared with wild-type plants after yellow leaf curl virus (TYLCV) infection, highlighting the function of PDI in maintaining redox homeostasis in response to TYLCV infection (Li *et al.*, 2020). It should also be noted that as a key regulator of systemic acquired resistance (SAR), NPR1 (non-expressor of pathogenesis-related genes 1) also modulated the expression of ER-resident proteins, such as PDI, BiP2, calnexin, and antioxidant enzymes (Wang *et al.*, 2005), suggesting a potential correlation between UPR, SAR, and antioxidant capacity. Importantly, Brandizzi’s lab elucidated that ER stress-induced redox activation of NPR1 could suppress the transcriptional activity of bZIP28 and bZIP60 in the UPR independent of salicylic acid (SA), further interwining the crosstalk between redox status and the UPR (Lai *et al.*, 2018).

### Newly identified proteases cleaving membrane-anchored TFs

The timely activation of stress-responsive genes is crucial for plants, which depends on the translocation of the ER membrane-anchored TFs to the nucleus for transcriptional reprogramming of pro-survival or pro-death genes (Ko and Brandizzi, 2024). The release of these TFs requires cleavage by certain proteases; however, these proteases have still not been systematically explored.

Previous studies have indicated that the bZIP28 release from the ER membrane upon ER stress is mediated by the sequential cleavage by site-1 protease (S1P) and S2P. However, this is still a matter of controversy. Sun *et al.* (2015) demonstrated that the RRIL^573^ site was essential for proteolytic processing and nuclear translocation of bZIP28 in response to ER stress. Iwata *et al*. (2017) provided genetic evidence that bZIP28 failed to accumulate in the nucleus in *s2p* mutants upon tunamycin treatment. Concurrently, aberrant ER stress-responsive gene expression and higher sensitivity to tunicamycin were observed in the *s2p* mutants. Moreover, two membrane-anchored bZIP28 proteins with a shorter C-terminus were detected in the *s2p* mutants. The authors proposed that proteolytic activation of bZIP28 under ER stress might be implemented through unidentified protease(s) and S2P, rather than S1P.

In addition to S1P and S2P, rhomboid proteases and signal peptide peptidases have also been implicated in this intramembrane proteolysis upon ER stress. For instance, hypoxia induced the release of ANAC013 from the ER membrane, which required the action of the RHOMBOID-LIKE 2 (RBL2) protease (Eysholdt-Derzsó *et al.*, 2023). The subsequent activation of hypoxia core genes depends on the recognition of the mitochondrial dysfunction motif (MDM) in their promoters. However, ANAC016 and ANAC017 failed to translocate into the nucleus despite possessing transactivation and MDM motif recognition abilities. Further investigation is required to elucidate the role of the mitochondrial retrograde signal promoting ANAC013 cleavage by RBL2. Indeed, analogous cases have been reported in mammalian cells. The signal peptidase complex cleaved iRhom2 pseudoprotease, leading to translocation of its N-terminal cytoplasmic domain to the nucleus, thereby affecting gene expression and causing skin pathologies (Dulloo *et al.*, 2024). Notably, evidence suggested that *Plasmodiophora brassicae*, *Agrobacterium tumefaciens*, and *B. cinerea* infections may induce an increased respiration rate, or an abrupt drop in O_2_ concentration in tissues, resulting in hypoxia (Zhao *et al*., 2012; Gravot *et al*., 2016; Valeri *et al.*, 2021). It would be intriguing to ascertain whether pathogens commonly exploit the ER homeostasis machinery by inducing local hypoxia, as reported for *B. cinerea* (Valeri *et al.*, 2021).

### State-of-the-art microscopy resolves ER dynamics in response to stress factors

The ER, with its extensive surface area, forms a network of ER tubules and cisternae (Stefano and Brandizzi, 2018). It also interacts with other organelles through membrane-contacting sites, facilitating communications (Wang *et al.*, 2017; Bian *et al.*, 2023). Notably, ER tubules and cisternae undergo continuous remodeling. Advanced microscopy has significantly deepened our insights into ER dynamics. Zhang *et al.* (2024, Preprint) applied real-time super-resolution imaging (structured illumination microscopy, SIM) to track and analyze ER dynamics. They utilized Swint ResU-Net, a U-shaped network that integrates Swin Transformer modules and residual modules, to automatically segment ER structures, identify growing tips, a three-way junction, a multi-way junction, and other structural features of ER, thereby enhancing the ability to distinguish various ER morphologies under stress conditions (Zhang *et al.*, 2024, Preprint). Similarly, VESICLE-ASSOCIATED MEMBRANE PROTEIN (VAMP)-ASSOCIATED PROTEIN 27-1 (VAP27-1), an ER–plasma membrane contact site (EPCS)-tethering protein, is enriched at EPCSs (Wang *et al.*, 2016). Man *et al.* (2024) tracked EPCS dynamics characterized by VAP27-1–enhanced green fluorescent protein (EGFP) using total internal reflection fluorescence (TIRF)-SIM imaging and single particle tracking, demonstrating that ER stress-induced VAP27-1 rearrangement contributed to resistance to ER stress. Moreover, the *vap27-1/3/4* triple mutant exhibited a severe cell death phenotype as compared with the wild-type seedling, suggesting that this phenotype may result from aberrant endocytosis, increased [Ca^2+^]_cyt_ influx, and autophagy defects, leading to higher sensitivity to ER stress (Man *et al.*, 2024). Indeed, VAPs deliver ATG8 to the ER membrane and maintain the EPCS, contributing to ER stress-induced ER-phagy (Zhao *et al.*, 2020). These studies unraveled the selectivity in ER-phagy, established a functional connection between UPR and ER-phagy, and defined the role of EPCSs between ER and the plasma membrane in ER-phagy.

## Concluding remarks and perspective

Although new players for sustaining ER homeostasis in response to pathogen invasion have been successively identified, there are still some unsolved issues in host–pathogen interaction, warranting further investigation.

(i) Effectors typically lack conserved functional domains, posing difficulties in elucidating their mechanisms. However, advancements in protein structure analysis, including cryo-electron microscopy and Alphafold2, have facilitated a comprehensive analysis of effector structures, potentially aiding in understanding their immune suppression mechanisms and rational design of resistance genes.
(ii) The molecular signatures from pathogens that activate HLP1 and other ER-phagy receptors remain elusive. Are these signals conserved across diverse pathogens? Moreover, apart from HLP1 and other HVA22 family members, are there additional sensors in the ER stress triggered by pathogens? Future endeavors to identify and manipulate these components may provide deeper insights into the machinery regulating cell fate decisions under biotic stress.
(iii) As some ER stress regulators, such as IRE1 and BiPs, are highly conserved across various cell types under diverse stress conditions, could moderate manipulation of these regulators induce UPRs, thereby conferring resistance to pathogens? This hinges on a thorough examination of the activation thresholds for UPR and prevention of unmitigated proteotoxic stress.
(iv) High-resolution microscopic techniques hold promise in providing detailed insights into ER morphology dynamics. It will also be intriguing to determine the precise thresholds for triggering unresolvable ER stress and even ER fragmentation.

Box 1. **Key developments in the understanding of endoplasmic reticulum homeostasis in plant–pathogen interactions**

- Meng *et al*. (2022) identified that a rice HVA22 family protein, OsHLP1, functioned in maintaining the normal ER tubule network. They found that *Magnaporthe oryzae* infection impaired ER homeostasis and degraded the OsHLP1-interacting immune regulator OsNTL6.
- Ko *et al*. (2022) identified the G-class bZIP TF2 (GBF2) and the *cis*-regulatory element G-box as plant UPR regulators. They demonstrated that GBF2 repressed UPR gene expression by competing with bZIP28 and bZIP60 at the G-box.
- Ko *et al*. (2023) showed PHOSPHATASE TYPE 2CA (PP2CA)-INTERACTING RING FINGER PROTEIN 1 to be a plant-speciﬁc regulator, balancing the UPR and UPS by modulating ABI5 stability.
- Liang *et al*. (2023) reported that OsHLP1, an ER-phagy receptor, recruited ER-located OsNTL6 for autophagic degradation by interacting with OsATG8b, subsequently activating disease resistance.
- Li *et al*. (2024) revealed that the powdery mildew effectors AVR_A1_ and BEC1016 targeted the ER J-domain protein *Hv*ERdj3B, an ERQC component, thus providing an evolutionary benefit to the powdery mildew.

## References

[CIT0001] Bao Y , Howell SH. 2017. The unfolded protein response supports plant development and defense as well as responses to abiotic stress. Frontiers in Plant Science 8, 344. 28360918 10.3389/fpls.2017.00344 PMC5350557

[CIT0002] Bao Y , Pu Y, Yu X, Gregory BD, Srivastava R, Howell SH, Bassham DC. 2018. IRE1B degrades RNAs encoding proteins that interfere with the induction of autophagy by ER stress in *Arabidopsis thaliana*. Autophagy 14, 1562–1573. 29940799 10.1080/15548627.2018.1462426 PMC6135571

[CIT0003] Bian JH , Su X, Yuan XY, Zhang Y, Lin JX, Li XJ. 2023. ER–membrane contact sites: cross talk between membrane-bound organelles in plant cells. Journal of Experimental Botany 74, 2956–2967. 36847172 10.1093/jxb/erad068

[CIT0004] Brandizzi F. 2021. Maintaining the structural and functional homeostasis of the plant endoplasmic reticulum. Developmental Cell 56, 919–932. 33662257 10.1016/j.devcel.2021.02.008 PMC8922286

[CIT0005] Breeze E , Vale V, McLellan H, Pecrix Y, Godiard L, Grant M, Frigerio L. 2023. A tell tail sign: a conserved C-terminal tail-anchor domain targets a subset of pathogen effectors to the plant endoplasmic reticulum. Journal of Experimental Botany 74, 3188–3202. 36860200 10.1093/jxb/erad075 PMC10199128

[CIT0006] Carrillo R , Iwai K, Albertson A, Dang G, Christopher DA. 2024. Protein disulfide isomerase-9 interacts with the lumenal region of the transmembrane endoplasmic reticulum stress sensor kinase, IRE1, to modulate the unfolded protein response in *Arabidopsis*. Frontiers in Plant Science 15, 1389658. 38817940 10.3389/fpls.2024.1389658 PMC11137178

[CIT0007] Deng Y , Humbert S, Liu JX, Srivastava R, Rothstein SJ, Howell SH. 2011. Heat induces the splicing by IRE1 of a mRNA encoding a transcription factor involved in the unfolded protein response in Arabidopsis. Proceedings of the National Academy of Sciences, USA 108, 7247–7252. 10.1073/pnas.1102117108 PMC3084119 21482766

[CIT0008] Ding Z , Zhang Z, Zhong J, Luo D, Zhou J, Yang J, Xiao L, Shu D, Tan H. 2016. Comparative transcriptome analysis between an evolved abscisic acid-overproducing mutant *Botrytis cinerea* TBC-A and its ancestral strain *Botrytis cinerea* TBC-6. Scientific Reports 6, 37487. 27892476 10.1038/srep37487 PMC5124961

[CIT0009] Dou D , Kale SD, Wang X, Jiang RH, Bruce NA, Arredondo FD, Zhang X, Tyler BM. 2008. RXLR-mediated entry of *Phytophthora sojae* effector Avr1b into soybean cells does not require pathogen-encoded machinery. The Plant Cell 20, 1930–1947. 18621946 10.1105/tpc.107.056093 PMC2518231

[CIT0010] Dulloo I , Tellier M, Levet C, Chikh A, Zhang B, Blaydon DC, Webb CM, Kelsell DP, Freeman M. 2024. Cleavage of the pseudoprotease iRhom2 by the signal peptidase complex reveals an ER-to-nucleus signaling pathway. Molecular Cell 84, 277–292.e9. 38183983 10.1016/j.molcel.2023.12.012 PMC7618786

[CIT0011] Eletto D , Chevet E, Argon Y, Appenzeller-Herzog C. 2014. Redox controls UPR to control redox. Journal of Cell Science 127, 3649–3658. 25107370 10.1242/jcs.153643

[CIT0012] Eysholdt-Derzsó E , Renziehausen T, Frings S, et al 2023. Endoplasmic reticulum-bound ANAC013 factor is cleaved by RHOMBOID-LIKE 2 during the initial response to hypoxia in *Arabidopsis thaliana*. Proceedings of the National Academy of Sciences, USA 120, e2221308120. 10.1073/pnas.2221308120 PMC10242721 36897975

[CIT0013] Fan G , Yang Y, Li T, Lu W, Du Y, Qiang X, Wen Q, Shan W. 2018. A *Phytophthora capsici* RXLR effector targets and inhibits a plant PPIase to suppress endoplasmic reticulum-mediated immunity. Molecular Plant 11, 1067–1083. 29864524 10.1016/j.molp.2018.05.009

[CIT0014] Fatima K , Naqvi F, Younas H. 2021. A review: molecular chaperone-mediated folding, unfolding and disaggregation of expressed recombinant proteins. Cell Biochemistry and Biophysics 79, 153–174. 33634426 10.1007/s12013-021-00970-5

[CIT0015] Gravot A , Richard G, Lime T, et al 2016. Hypoxia response in Arabidopsis roots infected by Plasmodiophora brassicae supports the development of clubroot. BMC Plant Biology 16, 251. 27835985 10.1186/s12870-016-0941-y PMC5106811

[CIT0016] Heimel K , Freitag J, Hampel M, Ast J, Bölker M, Kämper J. 2013. Crosstalk between the unfolded protein response and pathways that regulate pathogenic development in *Ustilago maydis*. The Plant Cell 25, 4262–4277. 24179126 10.1105/tpc.113.115899 PMC3877826

[CIT0017] Houck SA , Ren HY, Madden VJ, Bonner JN, Conlin MP, Janovick JA, Conn PM, Cyr DM. 2014. Quality control autophagy degrades soluble ERAD-resistant conformers of the misfolded membrane protein GnRHR. Molecular Cell 54, 166–179. 24685158 10.1016/j.molcel.2014.02.025 PMC4070183

[CIT0018] Iwata Y , Ashida M, Hasegawa C, Tabara K, Mishiba K-I, Koizumi N. 2017. Activation of the Arabidopsis membrane-bound transcription factor bZIP28 is mediated by site-2 protease, but not site-1 protease. The Plant Journal 91, 408–415. 28407373 10.1111/tpj.13572

[CIT0019] Jing M , Guo B, Li H, et al 2016. A *Phytophthora sojae* effector suppresses endoplasmic reticulum stress-mediated immunity by stabilizing plant binding immunoglobulin proteins. Nature Communications 7, 11685. 10.1038/ncomms11685 PMC4895818 27256489

[CIT0020] Jing MF , Wang YC. 2020. Plant pathogens utilize effectors to hijack the host endoplasmic reticulum as part of their infection strategy. Engineering 6, 500–504.

[CIT0021] Ko DK , Brandizzi F. 2022. Transcriptional competition shapes proteotoxic ER stress resolution. Nature Plants 8, 481–490. 35577961 10.1038/s41477-022-01150-w PMC9187302

[CIT0022] Ko DK , Brandizzi F. 2024. Dynamics of ER stress-induced gene regulation in plants. Nature Reviews. Genetics 25, 513–525. 10.1038/s41576-024-00710-4 PMC11186725 38499769

[CIT0023] Ko DK , Kim JY, Thibault EA, Brandizzi F. 2023. An IRE1–proteasome system signalling cohort controls cell fate determination in unresolved proteotoxic stress of the plant endoplasmic reticulum. Nature Plants 9, 1333–1346. 37563456 10.1038/s41477-023-01480-3 PMC10481788

[CIT0024] Lai Y-S , Renna L, Yarema J, Ruberti C, He SY, Brandizzi F. 2018. Salicylic acid-independent role of NPR1 is required for protection from proteotoxic stress in the plant endoplasmic reticulum. Proceedings of the National Academy of Sciences, USA 115, E5203–E5212. 10.1073/pnas.1802254115 PMC5984531 29760094

[CIT0025] Li T , Wang Y-H, Huang Y, Liu J-X, Xing G-M, Sun S, Li S, Xu Z-S, Xiong A-S. 2020. A novel plant protein-disulfide isomerase participates in resistance response against the TYLCV in tomato. Planta 252, 25. 32681182 10.1007/s00425-020-03430-1

[CIT0026] Li ZX , Howell SH. 2021. Review: the two faces of IRE1 and their role in protecting plants from stress. Plant Science 303, 110758. 33487343 10.1016/j.plantsci.2020.110758

[CIT0027] Li ZZ , Velásquez-Zapata V, Elmore JM, et al 2024. Powdery mildew effectors AVRA1 and BEC1016 target the ER J-domain protein HvERdj3B required for immunity in barley. Molecular Plant Pathology 25, e13463. 38695677 10.1111/mpp.13463 PMC11064805

[CIT0028] Liang YB , Meng FW, Zhao X, He XY, Liu J. 2023. OsHLP1 is an endoplasmic-reticulum-phagy receptor in rice plants. Cell Reports 42, 113480. 38019652 10.1016/j.celrep.2023.113480

[CIT0029] Liu RJ , Xia R, Xie Q, Wu YR. 2021. Endoplasmic reticulum-related E3 ubiquitin ligases: key regulators of plant growth and stress responses. Plant Communications 2, 100186. 34027397 10.1016/j.xplc.2021.100186 PMC8132179

[CIT0030] Lu X , Kracher B, Saur IM, Bauer S, Ellwood SR, Wise R, Yaeno T, Maekawa T, Schulze-Lefert P. 2016. Allelic barley MLA immune receptors recognize sequence-unrelated avirulence effectors of the powdery mildew pathogen. Proceedings of the National Academy of Sciences, USA 113, E6486–E6495. 10.1073/pnas.1612947113 PMC5081590 27702901

[CIT0031] Man Y , Zhang Y, Chen LH, Zhou JH, Bu YF, Zhang X, Li XJ, Li Y, Jing YP, Lin JX. 2024. The VAMP-associated protein VAP27-1 plays a crucial role in plant resistance to ER stress by modulating ER–PM contact architecture in Arabidopsis. Plant Communications 5, 100929. 38678366 10.1016/j.xplc.2024.100929 PMC11287176

[CIT0032] McLellan H , Boevink PC, Armstrong MR, Pritchard L, Gomez S, Morales J, Whisson SC, Beynon JL, Birch PRJ. 2013. An RxLR effector from *Phytophthora infestans* prevents re-localisation of two plant NAC transcription factors from the endoplasmic reticulum to the nucleus. PLoS Pathogens 9, e1003670. 24130484 10.1371/journal.ppat.1003670 PMC3795001

[CIT0033] Meng F , Zhao Q, Zhao X, et al 2022. A rice protein modulates endoplasmic reticulum homeostasis and coordinates with a transcription factor to initiate blast disease resistance. Cell Reports 39, 110941. 35705042 10.1016/j.celrep.2022.110941

[CIT0034] Nawkar GM , Lee ES, Shelake RM, Park JH, Ryu SW, Kang CH, Lee SY. 2018. Activation of the transducers of unfolded protein response in plants. Frontiers in Plant Science 9, 214. 29515614 10.3389/fpls.2018.00214 PMC5826264

[CIT0035] Nekrasov V , Li J, Batoux M, et al 2009. Control of the pattern-recognition receptor EFR by an ER protein complex in plant immunity. The EMBO Journal 28, 3428–3438. 19763086 10.1038/emboj.2009.262 PMC2776097

[CIT0036] Pastor-Cantizano N , Angelos ER, Ruberti C, Jiang T, Weng XY, Reagan BC, Haque T, Juenger TE, Brandizzi F. 2024. Programmed cell death regulator BAP2 is required for IRE1-mediated unfolded protein response in Arabidopsis. Nature Communications 15, 5804. 10.1038/s41467-024-50105-6 PMC11237027 38987268

[CIT0037] Pastor-Cantizano N , Ko DK, Angelos E, Pu Y, Brandizzi F. 2020. Functional diversification of ER stress responses in Arabidopsis. Trends in Biochemical Sciences 45, 123–136. 31753702 10.1016/j.tibs.2019.10.008 PMC6980780

[CIT0038] Praznik A , Fink T, Franko N, Lonzarić J, Benčina M, Jerala N, Plaper T, Roškar S, Jerala R. 2022. Regulation of protein secretion through chemical regulation of endoplasmic reticulum retention signal cleavage. Nature Communications 13, 1323. 10.1038/s41467-022-28971-9 PMC8904541 35260576

[CIT0039] Schmitz L , Schwier MA, Heimel K. 2019. The unfolded protein response regulates pathogenic development of *Ustilago maydis* by Rok1-dependent inhibition of mating-type signaling. mBio 10, e02756-19. 31848283 10.1128/mBio.02756-19 PMC6918084

[CIT0040] Shan L , He P, Li J, Heese A, Peck SC, Nürnberger T, Martin GB, Sheen J. 2008. Bacterial effectors target the common signaling partner BAK1 to disrupt multiple MAMP receptor-signaling complexes and impede plant immunity. Cell Host & Microbe 4, 17–27. 18621007 10.1016/j.chom.2008.05.017 PMC2830012

[CIT0041] Simoni EB , Oliveira CC, Fraga OT, Reis PAB, Fontes EPB. 2022. Cell death signaling from endoplasmic reticulum stress: plant-specific and conserved features. Frontiers in Plant Science 13, 835738. 35185996 10.3389/fpls.2022.835738 PMC8850647

[CIT0042] Stefano G , Brandizzi F. 2018. Advances in plant ER architecture and dynamics. Plant Physiology 176, 178–186. 28986423 10.1104/pp.17.01261 PMC5761816

[CIT0043] Stephani M , Picchianti L, Gajic A, et al 2020. A cross-kingdom conserved ER-phagy receptor maintains endoplasmic reticulum homeostasis during stress. eLife 9, e58396. 32851973 10.7554/eLife.58396 PMC7515635

[CIT0044] Sun J , Wang W, Zheng H. 2022. ROOT HAIR DEFECTIVE3 is a receptor for selective autophagy of the endoplasmic reticulum in Arabidopsis. Frontiers in Plant Science 13, 817251. 35283874 10.3389/fpls.2022.817251 PMC8907713

[CIT0045] Sun L , Zhang SS, Lu SJ, Liu JX. 2015. Site-1 protease cleavage site is important for the ER stress-induced activation of membrane-associated transcription factor bZIP28 in Arabidopsis. Science China. Life Sciences 58, 270–275. 25634523 10.1007/s11427-015-4807-6

[CIT0046] Taiz L , Zeiger E. 2002. Plant physiology, 3rd edn. Sunderland, MA: Sinauer Associates, 10–13.

[CIT0047] Valeri MC , Novi G, Weits DA, Mensuali A, Perata P, Loreti E. 2021. *Botrytis cinerea* induces local hypoxia in Arabidopsis leaves. New Phytologist 229, 173–185. 32124454 10.1111/nph.16513 PMC7754360

[CIT0048] Verchot J , Pajerowska-Mukhtar KM. 2021. UPR signaling at the nexus of plant viral, bacterial, and fungal defenses. Current Opinion in Virology 47, 9–17. 33360330 10.1016/j.coviro.2020.11.001

[CIT0049] Wang D , Weaver ND, Kesarwani M, Dong X. 2005. Induction of protein secretory pathway is required for systemic acquired resistance. Science 308, 1036–1040. 15890886 10.1126/science.1108791

[CIT0050] Wang GB , Zhao HN, Zou J, Liang WX, Zhao ZJ, Li DL. 2024. Role of BcSfb3, the subunit of COPII vesicles, in fungal development and pathogenicity, ER-phagy and autophagy in the gray mold fungus *Botrytis cinerea*. International Journal of Biological Macromolecules 263, 130379. 38403214 10.1016/j.ijbiomac.2024.130379

[CIT0051] Wang HX , Wang SM, Wang W, Xu L, Welsh LRJ, Gierlinski M, Whisson SC, Hemsley PA, Boevink PC, Birch PRJ. 2023. Uptake of oomycete RXLR effectors into host cells by clathrin-mediated endocytosis. The Plant Cell 35, 2504–2526. 36911990 10.1093/plcell/koad069 PMC10291037

[CIT0052] Wang JY , Dhroso A, Liu XL, Baum TJ, Hussey RS, Davis EL, Wang XH, Korkin D, Mitchum MG. 2021. Phytonematode peptide effectors exploit a host post-translational trafficking mechanism to the ER using a novel translocation signal. New Phytologist 229, 563–574. 32569394 10.1111/nph.16765

[CIT0053] Wang PW , Hawes C, Hussey PJ. 2017. Plant endoplasmic reticulum–plasma membrane contacts sites. Trends in Plant Science 22, 289–297. 27955928 10.1016/j.tplants.2016.11.008

[CIT0054] Wang PW , Hawkins TJ, Richardson C, Sparkes I, Hawes C, Hussey PJ. 2016. Plant VAP proteins: domain characterization, intercellular localization, and role in plant development. New Phytologist 210, 1311–1326. 27159525 10.1111/nph.13857

[CIT0055] Wu J , Michaeli S, Picchianti L, Dagdas Y, Galili G, Peled-Zehavi H. 2021. ATI1 (ATG8-interacting protein 1) and ATI2 define a plant starvation-induced reticulophagy pathway and serve as MSBP1/MAPR5 cargo receptors. Autophagy 17, 3375–3388. 33487099 10.1080/15548627.2021.1872886 PMC8632275

[CIT0056] Wu Z , Newstead S, Biggin PC. 2020. The KDEL trafficking receptor exploits pH to tune the strength of an unusual short hydrogen bond. Scientific Reports 10, 16903. 33037300 10.1038/s41598-020-73906-3 PMC7547670

[CIT0057] Zhang X , Ding X, Marshall RS, Paez-Valencia J, Lacey P, Vierstra RD, Otegui MS. 2020. Reticulon proteins modulate autophagy of the endoplasmic reticulum in maize endosperm. eLife 9, e51918. 32011236 10.7554/eLife.51918 PMC7046470

[CIT0058] Zhang YH , Liu JZ, Sun ZZ, et al 2024. Cell type specific responses of the endoplasmic reticulum dynamics to environmental stress. bioRxiv doi: https://doi.org/10.1101/2024.01.23.576814. [Preprint].

[CIT0059] Zhao D , Zou CX, Liu XM, Jiang ZD, Yu ZQ, Suo F, Du TY, Dong MQ, He WZ, Du LL. 2020. A UPR-induced soluble ER-phagy receptor acts with VAPs to confer ER stress resistance. Molecular Cell 79, 963–977. 32735772 10.1016/j.molcel.2020.07.019

[CIT0060] Zhao Y , Wei T, Yin KQ, Chen Z, Gu H, Qu LJ, Qin G. 2012. Arabidopsis RAP2.2 plays an important role in plant resistance to *Botrytis cinerea* and ethylene responses. New Phytologist 195, 450–460. 22530619 10.1111/j.1469-8137.2012.04160.x

